# Evaluating the Effectiveness of an E-Mental Health Intervention for People Living in Lebanon: Protocol for Two Randomized Controlled Trials

**DOI:** 10.2196/21585

**Published:** 2021-01-28

**Authors:** Edith van 't Hof, Eva Heim, Jinane Abi Ramia, Sebastian Burchert, Ilja Cornelisz, Pim Cuijpers, Rabih El Chammay, Melissa Harper Shehadeh, Philip Noun, Filip Smit, Chris van Klaveren, Mark van Ommeren, Edwina Zoghbi, Kenneth Carswell

**Affiliations:** 1 Department of Mental Health and Substance Use World Health Organization Geneva Switzerland; 2 Department of Psychology Psychopathology and Clinical Intervention University of Zurich Zurich Switzerland; 3 National Mental Health Programme Ministry of Public Health of Lebanon Beirut Lebanon; 4 Department of Education and Psychology Division of Clinical Psychological Intervention Freie Universität Berlin Berlin Germany; 5 Department of Educational and Family Studies Amsterdam Center for Learning Analytics Vrije Universiteit Amsterdam Netherlands; 6 Department of Clinical, Neuro and Developmental Psychology Amsterdam Public Health Research Institute Vrije Universiteit Amsterdam Netherlands; 7 Department of Digital Health and Innovations World Health Organization Geneva Switzerland; 8 Country Office for Lebanon World Health Organization Beirut Lebanon

**Keywords:** e-mental health, psychological interventions, guided-self-help, global mental health, adversity, Lebanon, Syrians

## Abstract

**Background:**

The lack of availability of evidence-based services for people exposed to adversity globally has led to the development of psychological interventions with features that will likely make them more scalable. The evidence for the efficacy of e-mental health from high-income countries is compelling, and the use of these interventions could be a way to increase the coverage of evidence-based psychological interventions in low- and middle-income countries. Step-by-Step is a brief (5-session) intervention proposed by the World Health Organization as an innovative approach to reducing the suffering and disability associated with depression.

**Objective:**

This study aims to evaluate the effectiveness and cost-effectiveness of a locally adapted version of Step-by-Step with Syrian nationals (trial 1) and Lebanese nationals and other populations residing in Lebanon (trial 2).

**Methods:**

This Step-by-Step trial involves 2 parallel, two-armed, randomized controlled trials comparing the e-intervention Step-by-Step to enhanced care as usual in participants with depressive symptoms and impaired functioning. The randomized controlled trials are designed and powered to detect effectiveness in 2 populations: Syrians in Lebanon (n=568) and other people residing in Lebanon (n=568; Lebanese nationals and other populations resident in Lebanon). The primary outcomes are depressive symptomatology (measured with the Patient Health Questionnaire-9) and functioning (measured with the World Health Organization Disability Assessment Scale 2.0). Secondary outcomes include anxiety symptoms, posttraumatic stress disorder symptoms, personalized measures of psychosocial problems, subjective well-being, and economic effectiveness. Participants are mainly recruited through online advertising. Additional outreach methods will be used if required, for example through dissemination of information through partner agencies and organizations. They can access the intervention on a computer, tablet, and mobile phone through a hybrid app. Step-by-Step has 5 sessions, and users are guided by trained nonspecialist “e-helpers” providing phone-based or message-based support for around 15 minutes a week.

**Results:**

The trials were funded in 2018. The study protocol was last verified June 20, 2019 (WHO ERC.0002797) and registered with ClinicalTrials.gov (NCT03720769). The trials started recruitment as of December 9, 2019, and all data collection was completed in December 2020.

**Conclusions:**

The Step-by-Step trials will provide evidence about the effectiveness of an e-mental health intervention in Lebanon. If the intervention proves to be effective, this will inform future scale-up of this and similar interventions in Lebanon and in other settings across the world.

**Trial Registration:**

ClinicalTrials.gov NCT03720769; https://clinicaltrials.gov/ct2/show/NCT03720769

**International Registered Report Identifier (IRRID):**

DERR1-10.2196/21585

## Introduction

Most people suffering from mental health problems do not access mental health care. It is estimated that in low- and middle-income countries, the majority (76%-85%) of people suffering from mental disorders receive no treatment at all [1]. This disparity is even bigger in communities exposed to adversities where the prevalence of mental health problems is higher and resources are often scarce [2]. The lack of availability of evidence-based services for people exposed to adversity globally has led to an interest in developing psychological interventions that are more likely to be scalable in low-resource settings [3].

Lebanon has a history of political instability that has negatively impacted the development of the country. There are approximately 1.5 million Syrian refugees in Lebanon, of which 74% are lacking legal status [4]. The country’s resources are extremely stretched, and the growing number of refugees puts extra pressure on the labor market and infrastructure. The treatment gap for mental health problems is estimated at over 90% by the National Mental Health Programme at the Ministry of Public Health (MoPH) [5]. A national epidemiological survey (involving 2857 people in Lebanon) published before the Syrian civil war showed that 1 in 6 people met the criteria for a mental disorder, with 27.0% of these “serious” [6]. Only 1 in 9 respondents with a mental disorder had ever obtained any treatment. More recent data show that the situation has improved, but treatment seeking still remains low, with a treatment gap of approximately 80% [7]. Affordable and accessible mental health care is very limited in Lebanon. MoPH data from 2015 show that there are 1.26 psychiatrists and 3.42 psychologists per 100,000 population and 97% of the mental health care staff works in private practice in Lebanon, which limits access to affordable care [5]. Since 2015, the MoPH in Lebanon has trained staff in more than 70 primary health care centers nationwide on the assessment and management of people with mental health conditions following World Health Organization (WHO) Mental Health Gap Action Programme (mhGAP) guidelines [8]. With a high prevalence of mental health problems in both refugees and the Lebanese host community and limited resources, there is a considerable need to scale up mental health and psychosocial support services in Lebanon.

E-mental health, which is the use of electronic devices to provide mental health interventions, could be a way to increase the coverage of evidence-based psychological interventions in a sustainable manner. In high-income countries, guided, online, self-help programs have been found to be as effective as the same interventions provided face-to-face [9-11]. In addition, such programs have been shown to reduce symptoms of mental disorders in routine care [12]. With ample evidence supporting the use of e-mental health, a number of countries now include e-mental health in their national mental health strategies and treatment guidelines (eg, the Netherlands, the United Kingdom, Australia, New Zealand, Scandinavian countries [13], and Lebanon [5,14]). In Lebanon, a substantial proportion of the population has access to mobile phones (92%) and the internet (78%) [15]. This suggests that many Lebanese have the means to access an e-health intervention, with this percentage likely to rise in the coming years [16]. The adult literacy rate is 88%, with youth literacy at 96% [17]. Syrians in Lebanon also have access to smartphones and the internet, with 80% reporting access at the household level (eg, 1 phone per household) [18]. The potential of digital interventions is that they can reach more people than more in-person interventions, which commonly have high dropout rates.

Step-by-Step is a brief (5-session) e-mental health intervention for depression proposed by the WHO as an innovative approach to reducing the suffering and disability associated with mental health issues [19]. It has been carefully designed and comprehensively adapted for use in Lebanon (including for refugees residing in Lebanon [20]). Step-by-Step is guided by trained nonspecialist “e-helpers” providing phone-based or message-based support to Step-by-Step users for around 15 minutes a week. It can also be used with different guidance models including no contact or contact on demand.

This paper describes the protocol for 2 randomized controlled trials (RCTs) of Step-by-Step in Lebanon. The study protocol has been informed by previous formative work [20,21], and an uncontrolled pilot [14] and a feasibility RCT, which has been submitted for publication, showed the acceptability and feasibility of Step-by-Step. The feasibility RCT was not powered to evaluate treatment effects, although estimates for complete cases at postassessment showed a significant reduction of depressive symptoms, anxiety, and posttraumatic stress and an improvement of functioning and well-being. The intention-to-treat (ITT) analysis showed a marginally nonsignificant effect on functioning and a trend in the expected direction for the other measures. These results indicate the importance of evaluating the effectiveness of Step-by-Step in fully powered RCTs. Because of the specific needs of different populations, the research effort is evaluating Step-by-Step with Syrians in Lebanon and other populations residing in Lebanon (eg, Lebanese and other refugee groups). Given the potentially substantial differences in experiences of migration and recent trauma between these 2 groups and that separate funding was received for the evaluations of Step-by-Step in these 2 groups, 2 RCTs were planned. The RCTs are powered to evaluate effectiveness and cost-effectiveness of Step-by-Step in the 2 populations. As the 2 RCTs use the same infrastructure and protocol, this paper describes both of them at the same time.

## Methods

### Objectives

This study aims to evaluate the effectiveness and cost-effectiveness of a locally adapted version of Step-by-Step in people residing in Lebanon, including Lebanese nationals, displaced Syrian people, and other populations resident in Lebanon. The RCTs will compare the e-intervention Step-by-Step to enhanced care as usual (ECAU) in participants with depressive symptoms and impaired functioning. One RCT will involve Syrian populations in Lebanon, and the other will involve other people residing in Lebanon.

### Design and Setting

The study is designed as 2 pragmatic, parallel, 2-arm RCTs. Participant-level outcomes will be measured at 3 time points: baseline (t0), posttreatment (t1), and follow-up (t2). Posttreatment (t1) has been set at 8 weeks after baseline (t0), and follow-up (t2) has been set at 3 months after posttreatment (t1), so 5 months after baseline (t0). Posttreatment is the primary outcome for the study. In both RCTs, Step-by-Step will be compared to ECAU in 1 RCT with Syrians in Lebanon (hereafter called “Syrian” group) and in the other RCT with other populations residing in Lebanon (eg, Lebanese and other refugee groups, hereafter called “Lebanese and others” group). Both RCTs use the exact same infrastructure, and the study is powered to detect effectiveness for both groups.

The primary hypothesis is that at posttreatment (8 weeks after baseline), in both the “Syrian” and “Lebanese and other” groups, people receiving Step-by-Step will have less severe depressive symptoms (measured with the Patient Health Questionnaire [PHQ-9]) and higher levels of functioning (measured with the World Health Organization Disability Assessment Scale [WHODAS] 2.0) compared to people receiving ECAU. The secondary hypothesis is that people receiving Step-by-Step will report fewer anxiety symptoms, posttraumatic stress disorder (PTSD) symptoms, and personally identified psychosocial problems as well as higher levels of subjective well-being. In addition, we hypothesize that offering Step-by-Step is more cost effective than ECAU.

### Study Arms

#### Step-by-Step intervention

Step-by-Step is brief (5 sessions) and has been designed to primarily address depressive symptoms [19]. Behavioral activation is the main active therapeutic agent in the intervention, as it is easy for users to engage with, has a very strong evidence base for depression [22,23], and can easily be adapted to a minimally guided, internet delivery model [24]. The intervention follows a narrative story–based approach to convey information together with illustrations and weekly exercises. The narrative and exercises aim to increase behavioral activation, including pleasurable activities and social support. Additional strategies to support this include 2 stress management techniques, a gratitude and positive self-talk exercise, and mood tracking where a user is regularly prompted to enter their mood on a graphical 5-point Likert scale. The story and its illustrations have been adapted to the local context, considering linguistic and cultural nuances within the different populations residing in Lebanon (broadly speaking, Lebanese, Syrian, and Palestinian people). Separate papers have been published with more information about the concept behind Step-by-Step [19] and about the adaptation process for Lebanon [20].

In brief, the Step-by-Step narrative has male and female versions with 2 versions per gender: 1 broadly for married people with children and 1 for younger single people without children. The text content is slightly adapted across these versions, but the therapeutic content stays the same. There are also different versions of the illustrations allowing people the choice between a bearded or unbearded character or a character with or without a headscarf. This accounts for gender differences and provides broad tailoring for the main cultural groups of target users. The language used in the intervention is simple so it can easily be understood, and the intervention has audio recordings of all the written text that can be played by people with lower levels of literacy. More information on the intervention can be found in Carswell et al [19] and Abi Ramia et al [20].

The intervention was first developed as an internet intervention and then developed into an iOS and Android app [21] that can be used mostly offline on mobile devices and into a web app that can be accessed via a web browser. These RCTs are testing Step-by-Step as a guided self-help intervention in which Step-by-Step is supported by trained nonspecialist research assistants (called “e-helpers”), who have weekly phone-based or message-based contact lasting approximately 15 minutes with users to provide support and guidance. E-helpers have an undergraduate degree in psychology or social work and work under the regular supervision of trained mental health practitioners. They will receive a 4.5-day training in the research protocols and the intervention itself, including how to guide users in implementing the techniques learned in the intervention and how to use the online system. They are supported in this by a manual and clear session-by-session outlines for the support contacts. Knowledge and therapeutic skills covered in the e-helper training include working with people with depression and other mental health problems, identifying and dealing with crisis situations, and responding to adverse events (AEs). E-helpers need to pass a competency test to be involved in the RCTs. During the trials, fidelity checks will be conducted to ensure adherence to the guidance protocol using a treatment fidelity checklist. The supervisor and the study coordinator will supervise up to 5% of the responsive support contacts through listening in to calls and reviewing messages.

Weekly clinical supervision will be provided to e-helpers by a clinical supervisor from MoPH with a good understanding of the Step-by-Step intervention and research project. Clinical supervision will ensure fidelity of the guidance provided and involves discussion of difficulties encountered in supporting the users of the intervention, as well as self-care for e-helpers. In addition, weekly supervision on research processes will be provided by the local study coordinator.

#### Enhanced Care as Usual (ECAU)

ECAU will consist of basic psychoeducation and referral to evidence-based care. If randomized into the ECAU condition, users will first receive basic psychoeducation on depression via the hybrid app. The text for the psychoeducational messages is taken from the first session of Step-by-Step to make sure the information provided is identical. After receiving the psychoeducation, ECAU users will receive a list of selected primary health care facilities with staff trained in the mhGAP where they can seek evidence-based care as usual consisting of assessment and pharmacological or psychosocial management of mental health conditions according to mhGAP [8].

### Randomization

Upon completion of the baseline assessment, participants will be randomized to either the intervention or ECAU, using a 1:1 allocation ratio. The randomization is handled by an algorithm for permuted block randomization that is built into the app and not accessible to the research team. The algorithm generates a random sequence of blocks with varying length. In each block, the number of seats for both groups is even, and the order is fully random.

### Sample Size Calculation

A recent meta-analysis of depression treatments in low- and middle-income countries has been conducted with 32 RCTs, looking at different intervention types, formats (eg, guided self-help, group therapy), and comparators (eg, waitlist, treatment as usual) [25]. An effect size of 0.73 (Hedges *g*) was found for symptoms of depression. Moreover, a recent meta-analysis of internet-based and mobile-based interventions for the treatment of depression in high-income countries, including 19 RCTs, showed an effect size of 0.90 (Hedges *g*) when comparing these treatments with the waitlist condition [26,27]. Despite these relatively high effect sizes reported in literature, the power calculation for these RCTs was completed for a more conservative (but still clinically significant) effect size of Cohen *d* = 0.5.

Assuming one primary outcome, power of 90%, and α of .05, the 2 RCTs need 85 participants in each of the 2 arms, in order to be able to detect a moderate effect size of 0.5. For the 2 primary outcomes considered, this yields a complete power (ie, the probability to detect statistically significant effects of at least 0.5 on both outcomes, given that both effects truly exist) ranging from 81% (independence between outcomes) to 90% (perfect correlation between outcomes). For individual power (ie, the probability of detecting an effect of 0.5 or larger for a particular outcome, given that the specific effect truly exists), applying a Bonferroni multiple testing procedure to control the family-wise error rate at or below an α of .05 yields an individual power of 84% [28]. We note that Bonferroni is overly conservative in the case of dependency between outcomes, such that the true family-wise error rate is likely to be somewhat below 5% [28].

In our feasibility RCT, we found a dropout rate of 70%, which is consistent with other e-mental health studies [29,30]. Accounting for a dropout rate of 70% [(2*85)/(1-0.70)=568], 568 displaced Syrian people and 568 other people residing in Lebanon will thus be recruited for the trials such that 85 are estimated to complete the intervention (complete 4 of 5 sessions) in the intervention arms of each trial.

### Participants

Any person aged over 18 years, residing in Lebanon, who can understand and speak Arabic or English, and has access to an internet-connected device is eligible to participate. Additional inclusion criteria are scoring 10 or above on the PHQ-9 [31] and scoring above 16 on the WHODAS for functional impairment [32]. Minors (under the age of 18 years) and people who have plans to end their life (as indicated by an answer of “yes” on an additional screening question - “In the past month, have you had serious thoughts or a plan to end your life?”) will be excluded from the study. Participants who answer “yes” to this additional screening question will be considered at imminent risk of suicide and will receive an on-screen message explaining that they may need additional mental health support with advice to go to an emergency room or call the national suicide hotline (Embrace Lifeline) established by the MoPH for suicide prevention. They will also be presented a list of facilities providing mhGAP care, encouraged to seek help, and provided with additional self-care tips.

### Procedures

**Figure 1:**
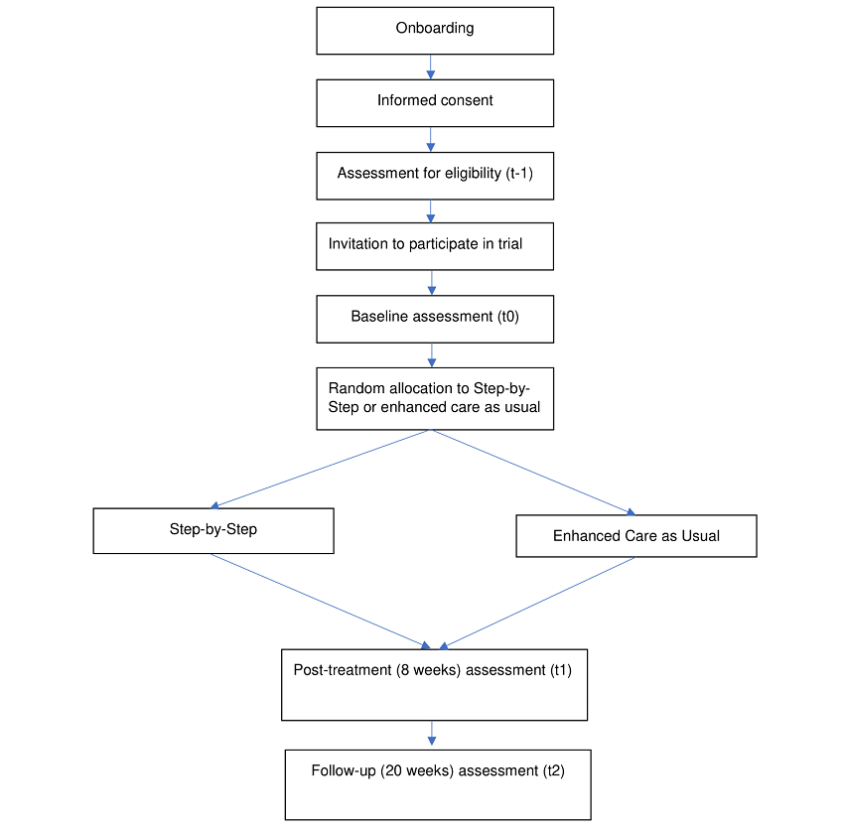
Flow chart of the study procedures.

The research procedures can be found in Figure 1. Recruitment of participants will be conducted through online advertising, primarily a social media campaign comprising of posts including videos, animations, gifs, and images. The posts will be disseminated through multiple channels, including the social media platforms of the National Mental Health Programme (NMHP) at the MoPH in Lebanon. The campaign will be conducted by a professional communication company in close collaboration with the NMHP team. Additional outreach methods will be used where required, for example, dissemination of posts through partner agencies and organizations.

People interested can access the website or download the native iOS or Android app version of Step-by-Step. They will enter the onboarding section of the app with information about Step-by-Step and the research project. The onboarding section of the app also contains an animation that explains the most important points in a short video.

People will apply to join the study by following on-screen instructions in the app or on the website. After giving consent, they will be asked to create an account. They will be asked to indicate their age and complete an initial self-screening measure (PHQ-9, WHODAS 2.0, and additional suicide screening question). If an individual meets the inclusion criteria, he or she is asked to complete the study baseline questionnaires. Upon completion of the baseline assessment, the individual will be randomized to either the intervention or ECAU, using a 1:1 allocation ratio in the program. If the individual does not score over the clinical cut-off on the PHQ-9, a box will appear saying that the intervention may not be a good fit for them and suggesting they seek support from a health care worker.

At sign-up, users will be asked to choose at least one method of follow-up contact from phone call, email, or SMS for reminders of the assessments. People allocated to Step-by-Step are also asked to confirm their contact preferences for regular support from e-helpers. Applicants will be able to contact a member of the study team throughout the self-screening and recruitment process for free, using telephone or messaging services. On completion of enrollment upon sign-up, research staff will either call or send a message to users in the control group (depending on their preferred contact option) to thank them for their participation in the study and to remind them of the format of the study (eg, posttreatment and follow-up questionnaires). Users who access the study through the app (ie, not via web browser) will be asked if they would like to receive notifications on their smartphone. These notifications will cover (1) assessments due (both conditions), (2) new sessions available (only intervention condition), (3) monthly automated messages to remind users of the upcoming assessments and to thank them for their ongoing participation in the study assessments (both conditions), and (4) mood tracking (only intervention condition). Users will receive an explanation at the beginning of the study about the purpose and reasons for these notifications and can opt out of some or all of the notifications at any time. If assessments are due, e-helpers will contact users (both intervention and control participants) via their preferred method of contact (eg, phone, email). As remuneration, users will receive US $20 phone credit for completion of all questionnaires at all time points.

### Informed Consent

All research participants will be asked for individual, electronic, informed consent. A known challenge in informed consent procedures in resource-limited settings is applicants feeling pressure to answer assessment questions because they see the research process as a route to access services and other resources. This problem is expected to be diminished by the fact that the recruitment will mainly be done through social media instead of face to face. All participants will go to the site on their own accord and be provided with detailed information at the start of the intervention. Before requesting consent, full information on the study will be provided in a video animation in the local language, which will also be available as both written and audio files, as part of the consent form. Applicants will receive information about what they can expect (ie, group allocation, assessments, reminders). In addition, they will be reminded that they are free to withdraw at any time and that nonparticipation will not affect, in any way, their access to usual health care.

E-helpers will receive in-depth training on the informed consent process and will be working in accordance with this protocol. All data and informed consent will be collected online, with telephone or messaging support from e-helpers if necessary. Respondents who decide to participate will be asked to electronically sign the consent form.

### Outcome Measures

#### Primary Outcomes

The primary outcomes are levels of depressive symptoms measured with the PHQ-9 [31] and levels of functioning measured with the WHODAS 2.0 [32] at posttreatment (8 weeks after baseline).

The PHQ-9 is a well-known 9-item instrument measuring presence and severity of depression [31]. As a severity measure, the PHQ-9 score may range from 0 to 27, since each of the 9 items can be scored from 0 (not at all) to 3 (nearly every day). The PHQ-9 has been validated in the Lebanese population with a cut-off score of 10 or above indicating moderate depression [33]. The PHQ-9 is now one of the main outcome measures worldwide that is used in research on psychological interventions and has been chosen as one of the core metrics for research on psychological interventions that should be included in all studies on psychological interventions for depression and distress funded by the Wellcome Trust, the National Institute of Mental Health, and other major funders [34].

The WHODAS 2.0 is a generic assessment instrument assessing health and disability [32]. It is used across all diseases, including mental, neurological, and substance use disorders, and in many global regions. It is simple to administer, is applicable across cultures, and can be used in all adult populations. WHODAS 2.0 covers 6 domains (cognition, mobility, self-care, getting along, life activities, participation). It assesses difficulties people have across these domains during the last 30 days. Difficulties are scored as none, mild, moderate, severe, or extreme.

#### Secondary Outcomes

**Figure 2:**
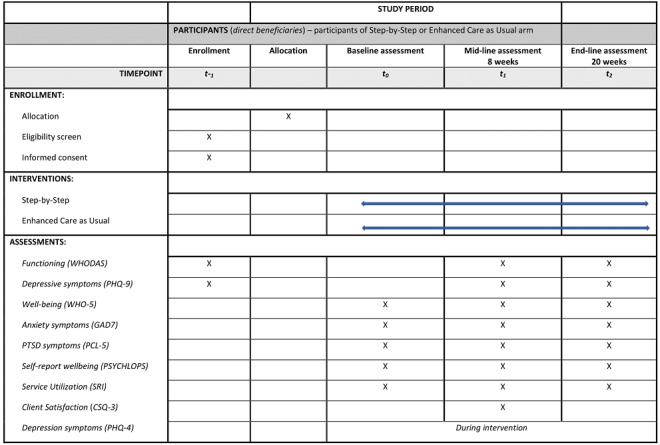
Schedule of enrollment, interventions, and assessments for Step-by-Step (SPIRIT figure). CSQ: Client Satisfaction Questionnaire 3; GAD: Generalized Anxiety Disorder; PCL-5: PTSD Checklist for DSM-5; PHQ: Patient Health Questionnaire; PSYCHLOPS: Psychological Outcomes Profile Instrument; SRI: Service Receipt Inventory; WHO: World Health Organization; WHODAS: World Health Organization Disability Assessment Scale.

Secondary outcomes include levels of subjective well-being (WHO-5) [35], levels of anxiety symptoms (Generalized Anxiety Disorder-7 [GAD-7]) [36], and levels of PTSD symptoms (PTSD Checklist for DSM-5 [PCL-5]) [37]. In addition, subjective problems are assessed using the Psychological Outcomes Profile Instrument (PSYCHLOPS) [38]. Satisfaction will be assessed using the 3-item version of the Client Satisfaction Questionnaire (CSQ) [39], and service utilization will be measured with an adapted version of the Client Service Receipt Inventory (CSRI) [40]. Please see Figure 2 for an overview of the different measures at different time points.

The WHO-5 Well-Being Index is a 5-item questionnaire measuring current psychological well-being and quality of life, rather than psychopathology [35]. Scores range from 0 to 25. The scale has demonstrated sensitivity to change in well-being and is available in multiple languages.

The GAD-7 is a 7-item self-report questionnaire for generalized anxiety disorder widely used in primary and specialist care as an indicator of anxiety symptoms [36]. It consists of Likert scale questions including items on nervousness, anxiety, restlessness, and fear. It is being included in this study as a means to investigate whether the intervention, which includes a stress reduction exercise and cognitive coping strategies, may reduce comorbid symptoms of anxiety. The GAD-7 has been validated in the Lebanese population [33].

PTSD symptoms during the past week will be measured using the abbreviated 8-item version of the PCL-5 [37]. Items are rated on a 5-point scale from 1 to 5 and add up to a total severity score of 30. The previous short version of the PCL (PCL-6) [41] that was based on the diagnostic criteria of DSM-IV has shown good psychometric properties and has been tested in diverse cultural settings, including Lebanon [42].

PSYCHLOPS [17] is a person-centered outcome measure consisting of 4 questions across 3 domains: problems (2 questions), functioning (1 question), and well-being (1 question). Participants are asked to indicate self-identified problems. Responses are scored on an ordinal 6-point scale producing a maximum score of 20 (5 points per question). The pre- and postintervention versions of PSYCHLOPS consist of the same 4 questions but the posttherapy version adds an overall evaluation question (determining self-rated outcome ranging from “much better” to “much worse”). PSYCHLOPS has been validated in primary care populations across several countries [20,21]. It is currently used in WHO studies in Pakistan, Kenya, and Uganda.

The CSQ [39] is an easily scored and administered 8-item measure that is designed to measure client satisfaction with mental health services. It includes an additional free response field as well as the 8 questions that are scored on a Likert scale. To decrease questionnaire burden, this study used the 3-item version of the CSQ [43].

The CSRI was developed for the collection of data on service utilization and related characteristics of people with mental disorders, as the basis for calculating the costs of care for mental health cost-effectiveness research [40]. The CSRI was adapted for online use within a large European Union–funded program, the STRENGTHS program [21], further adapted by the team for use in Lebanon and pilot-tested in the feasibility RCT.

During the course of the intervention, the PHQ-4 [44] will be used to monitor depressive symptoms on a weekly basis regardless of progress in the sessions. The 4-item version has shown good psychometric properties, and the Arabic version was validated among displaced Syrian people in Germany [44,45].

Data on sociodemographic information (sex, age, education, marital status, and work status) will be collected through questions A1-A5 of the 12-item self-report version of the WHODAS 2.0. In addition, users will be asked where they heard about the study.

### Data Management

Most data will be collected electronically in the app, and all electronic data will be stored on password-protected computers. The data collected through computers or devices will be downloaded from the platform and migrated into a data analysis software program. Hardcopies of data from the study (for example, from qualitative interviews and supervision notes) will be safely stored in locked cabinets. No personal data will be used in publications or presentations. The accuracy of data storage and output will be monitored bi-weekly by an external person.

Because of the nature of this innovative intervention and the data being collected online, considerable emphasis has been given to privacy and security of client data. Programming of the iOS and Android apps and web versions is being managed by Freie Universität Berlin. The intervention software and all procedures involving the software will be developed in compliance with the European Union General Data Protection Regulation.

### Data Analyses Plan

#### Statistical Analyses

For the 2 RCTs, both ITT analysis (including all randomized participants) and completers’ analyses (per protocol) will be carried out. First, the mean differences between the 2 treatment arms at baseline, postintervention, and 3-month follow-up will be determined. Then, the treatment effect will be estimated based on ITT analysis using regression estimation models with the principal predictor being treatment assignment status. Missing outcome observations for participants will be imputed using multiple imputation exploiting prescores and a set of prespecified background characteristics (gender, age, education, and severity of symptoms). Given that there are 2 primary outcomes of interest, we will impute using multivariate normal regression using an iterative Markov Chain Monte Carlo method based on initial treatment assignment. The aforementioned prespecified covariates and baseline measurement of primary endpoint will be added to the baseline model for improved precision. Potential bias concerns as a result of nonrandom missing outcome observations will be addressed by estimating Lee bounds. Then, 95% confidence intervals will be constructed, both for the regression-generated point estimates and Lee bounds [46] interval estimates. To further tighten confidence interval bounds in the context of differential and potentially nonrandom attrition between treatment arms, Random Forest Lee Bounds will be estimated (using the approach in Cornelisz et al [47]).

These treatment effect analyses will be performed for both primary outcome measures, PHQ-9 and WHODAS 2.0. Concerns of multiple testing error will be addressed by maintaining an experiment-wise type I error of 5%. In order to address potential heterogeneity, treatment effects will be estimated for subgroups (eg, based on prescores). Finally, average treatment effects on the treated will be estimated treatment and corresponding measures of clinically meaningful change, and numbers needed to treat (using the approach in Furukuwa et al [48]) will be explored.

In addition, the same analyses will be carried out for analyzing clinical outcomes measured at each assessment time: anxiety (GAD-7), wellbeing (WHO5), posttraumatic stress reactions (PCL-5, 8-item version), and self-identified symptoms (PSYCHLOPS).

#### Health Economic Analysis

The health economic evaluation will be conducted from the perspective of the health care system to determine the difference in costs over the difference in outcomes in the intervention arm as compared to the ECAU condition.

Costs include intervention cost (eg, costs for hosting and maintaining the intervention, costs for e-helpers) plus health care costs (eg, participants’ service use as assessed with the adapted CSRI). In addition, we will attempt to compute costs stemming from productivity losses owing to absenteeism and presenteeism (work cut back). Costs will be expressed in international dollars for the reference year 2019.

In the health economic evaluation, the central outcome will be treatment response defined as a pre-post symptom reduction of at least 50% on the PHQ with PHQ posttest scores below the cut-off of 10, thus indicating a clinically significant reduction in depressive symptoms. Treatment response will be defined in a similar way for WHODAS 2.0.

Costs, C, and effects, E, will combined in the incremental cost-effectiveness ratio (ICER) defined as (C_1_- C_0_)/(E_1_ – E_0_), where subscripts 1 and 0 refer to the intervention and ECAU conditions, respectively. The ICER can be interpreted as the additional cost per treatment responder.

Stochastic uncertainty in the ICER will be captured as a scatter of simulated ICERs over the ICER plane using 2500 bootstraps. For decision-making purposes, an ICER acceptability curve will be plotted indicating the likelihood that the intervention can be regarded as more cost-effective than the ECAU condition given a range of willingness-to-pay ceilings. Finally, sensitivity analyses will be directed at uncertainty in the main cost-drivers. The Consolidated Health Economic Evaluation Reporting Standards (CHEERS) guideline [49] will be followed when reporting the cost-effectiveness analysis.

### Qualitative Evaluation

To evaluate satisfaction with Step-by-Step, barriers and facilitators to adherence, and relevant information for scale-up, semistructured interviews will be conducted with a subsample of Step-by-Step participants (including both completers as well as noncompleters), control arm participants, e-helpers, supervisors, and other key informants. Up to 10 people per group will be interviewed. This number is based on previous experience of the number of participants needed to reach saturation. Data will be analyzed thematically. In addition, as part of process monitoring, a sample of session notes from e-helper records of their contacts with clients, as well as supervision records, will be reviewed and analyzed.

Informed consent will be reconfirmed from all participants immediately prior to interviews, and the interviews will take place face to face or over phone by trained interviewers. Interviewers will be using a draft semistructured interview guide with key questions that are identified for exploration, with additional prompt questions to fully explore each question in depth. Key areas that are explored include the general experience of using Step-by-Step, acceptability, feasibility, user satisfaction, and perceived effectiveness. Interviews will be conducted no longer than 4 weeks after the final outcome assessments and are expected to last no longer than 1 hour.

### Qualitative Data Analyses

The qualitative data collected from key informant interviews and notes during the process evaluation will be analyzed thematically. The transcribed and translated data will de coded in NVivo [50] by multiple raters, and interrater reliability will be calculated using Kappa scores. Qualitative data will be analyzed using thematic analysis and triangulation.

### Ethical Considerations

The intervention is based on evidence-based therapeutic techniques, and it is unlikely that distress will increase because of participation in the program. E-helpers will have access to the weekly depression scores (measured with the PHQ-4) of the participants and can therefore monitor distress levels over the course of the intervention. Participants who show symptom worsening (ie, a change in category from mild to moderate or moderate to severe) will be automatically flagged by the system. All involved research staff will be trained in communication skills, providing support, responding to distress, and procedures for AEs, including referral procedures.

All AEs and serious adverse events (SAEs) reported spontaneously by the participant or identified through study measures at any time will be recorded by the e-helpers. They will then notify the research coordinator and clinical supervisor for immediate follow-up action. All AEs will be followed up by the e-helper and the clinical supervisor on a regular basis. If, during self-screening or treatment, an AE should occur (eg, the participant discloses plans to end their life or there is a serious protection concern requiring assistance), e-helpers will assist the users by following protocols that are based on local pathways and laws in Lebanon. Any AEs and SAEs along with actions taken will be documented and reported to the local ethical review committee (ERC). SAEs will be reported within 24 hours (on working days) and AEs within 2 business days. The local ERC will review any SAEs as soon as possible and any AEs each month. They will determine any appropriate action with respect to ongoing study conduct. All SAEs will be reported to the WHO ERC.

Ethical approval has been received locally from Saint-Joseph’s University in Beirut (Protocol: CEHDF862) and by the WHO Ethical Review Committee (Version 7; Protocol ID: ERC.0002797).

## Results

The trials were funded in 2018. The study protocol (version 7) was last verified on June 20, 2019 (WHO ERC.0002797) and registered with ClinicalTrials.gov (NCT03720769). The trials started recruitment as of December 9, 2019, and all data collection was completed in December 2020. Subsequent protocol modifications will be reported to funders, institutional review boards, and registered with ClinicalTrials.gov.

## Discussion

Formative work [19-21], an open pilot [14], and a feasibility RCT preceded and informed this study protocol for RCTs to evaluate the effectiveness and cost-effectiveness of Step-by-Step in Lebanon. The RCTs will contribute to the evidence base for the potential of guided psychological self-help using task-shifting in low- and middle-income countries [3]. It will also contribute to the growing evidence base for the potential of digital interventions to reach broader populations with evidence-based care [51-53] and will address a gap in the evidence base in low- and middle-income countries [54].

Step-by-Step is an innovative approach to reducing the suffering and disability associated with psychological distress in a middle-income country, and after testing, WHO aims to release the intervention with adaptation and implementation guidance for use in other settings. However, merely testing the effectiveness of the intervention and releasing it as a public good will not be enough to ensure the intervention will reach people that need help and can benefit from it. After successfully testing the effectiveness of Step-by-Step in RCTs, a next step would be to study ways to implement the intervention outside of a research context and identify sustainable implementation models that will support scale-up of this intervention or similar interventions in Lebanon and other countries.

## Abbreviations

AE: adverse event
CHEERS: Consolidated Health Economic Evaluation Reporting Standards
CSQ: Client Satisfaction Questionnaire
CSRI: Client Service Receipt Inventory
ECAU: enhanced care as usual
ERC: ethical review committee
GAD-7: Generalized Anxiety Disorder-7
ICER: incremental cost-effectiveness ratio
ITT: intention-to-treat
mhGAP: Mental Health Gap Action Programme
MoPH: Ministry of Public Health
NMHP: National Mental Health Programme
PCL-5: PTSD Checklist for DSM-5
PHQ: Patient Health Questionnaire
PSYCHLOPS: Psychological Outcomes Profile Instrument
PTSD: posttraumatic stress disorder
RCT: randomized controlled trial
SAE: serious adverse event
WHO: World Health Organization
WHODAS: World Health Organization Disability Assessment Scale
